# GARFIELD, a toolkit for interpreting ultrafast electron diffraction data of imperfect quasi-single crystals

**DOI:** 10.1063/4.0000286

**Published:** 2025-04-25

**Authors:** Alexander Marx, Sascha W. Epp

**Affiliations:** Max Planck Institute for the Structure and Dynamics of Matter, CFEL, Luruper Chaussee 149, 22761 Hamburg, Germany

## Abstract

The analysis of ultrafast electron diffraction (UED) data from low-symmetry single crystals of small molecules is often challenged by the difficulty of assigning unique Laue indices to the observed Bragg reflections. For a variety of technical and physical reasons, UED diffraction images are typically of lower quality when viewed from the perspective of structure determination by single-crystal x-ray or electron diffraction. Nevertheless, time series of UED images can provide valuable insight into structural dynamics, providing that an adequate interpretation of the diffraction patterns can be achieved. Garfield is a collection of tools with a graphical user interface designed to facilitate the interpretation of diffraction patterns and to index Bragg reflections in challenging cases where other indexing tools are ineffective. To this end, Garfield enables the user to interactively create, explore, and optimize sets of parameters that define the diffraction geometry and characteristic properties of the sample.

## I. INTRODUCTION

Crystal structure determination by single-crystal x-ray or electron diffraction is usually based on the collection of a series of diffraction images, each containing a number of sharp and distinct reflections. Typically, the first step in diffraction data analysis is some form of “interpretation” of the diffraction patterns, which leads to an enormous reduction in the amount of data. The goal is to identify isolated reflection peaks and assign them to reciprocal lattice points (RLPs) of a perfect crystal with Laue indices *hkl* and further to use the measured (partial) intensities to obtain estimates of the underlying full Bragg intensities. These full intensities are—in the simplest cases—proportional to $|F_{\text{hkl}}{|}^{2}$, the squared moduli of the structure factors. The indexing task can be demanding, but powerful auto-indexing tools have been developed for various use cases and incorporated into commonly used crystallographic software packages.^1–16^ They all amount to some form of spatial or geometric analysis of tabulated peak positions. For large unit cells, a single diffraction pattern can be sufficient to determine the orientation of the diffracting crystal and uniquely assign the reflection indices, especially with prior knowledge of the unit cell. This is of great benefit for serial femtosecond crystallography (SFX) and serial synchrotron crystallography (SSX) of macromolecular crystals where thousands of diffraction images, each taken from a different crystal, can be processed in a short time and with a high success rate.^17–20^

Due to their small wavelength and strong interaction with matter, the diffraction of electrons opens up new possibilities, but it also presents new challenges. The contribution of multiple scattering to the diffraction signal can be significant, potentially necessitating a dynamical approach rather than a kinematical one. The samples must be sufficiently thin, typically on the order of 100 nanometers or less, depending on the atomic composition. Moreover, the intrinsic small wavelength renders the indexing of individual diffraction images more difficult, as each image only encompasses information from a nearly planar section through reciprocal space. To address these challenges, a range of innovative electron diffraction protocols have been developed in modern electron microscopy. The most crucial aspect is the limitation of the probe volume to thin and flawless regions of the sample through the use of selected area electron diffraction (SAED) or nanobeam diffraction (NBD). In recent years, notable advancements have been made in the field of crystal structure determination by “three-dimensional electron diffraction,”^21^ both in material science and macromolecular crystallography. The advent of new methods and automated protocols, including microcrystal electron diffraction (MicroED),^22^ precession electron diffraction (PED),^23^ and parallel NBD combined with STEM imaging, has considerably alleviated the adverse influences of radiation damage as well as multiple scattering and facilitated indexing. The continuous expansion of the range of single-crystal diffraction data that can be indexed automatically has made serial electron crystallography with nanocrystalline materials^24^ and protein nanocrystals^25^ a feasible undertaking.

Ultrafast electron diffraction of bright femtosecond electron pulses (UED)^26–34^ for the analysis of structural dynamics poses unique requirements for the interpretation of the diffraction data. The goal of a UED experiment is to study structural changes after perturbation of the sample, e.g., by a short laser pulse at time $t_{0}$, by recording a series of diffraction images at times $t_{i}=t_{0}+\Delta t_{i}$ and comparing them with images recorded without perturbation ( $\Delta t_{i}<0$). The data acquisition must be at a sufficient signal-to-noise level to allow accurate measurement of differences in diffraction intensities due to potentially small structural changes. UED data collected from thin, single-crystal samples typically consist of one or several time series of diffraction images, each series comprising a large number of nearly identical diffraction patterns. Thus, “indexing” one representative image per time series would be sufficient to index all members of the series. The challenges of interpreting such UED data often begin with the problem of assigning the correct Laue indices to the observed reflections since the observed data often do not look sufficiently similar to expectations derived from oversimplified approaches that are ultimately unsuitable for describing this type of diffraction data.

**FIG. 1.**
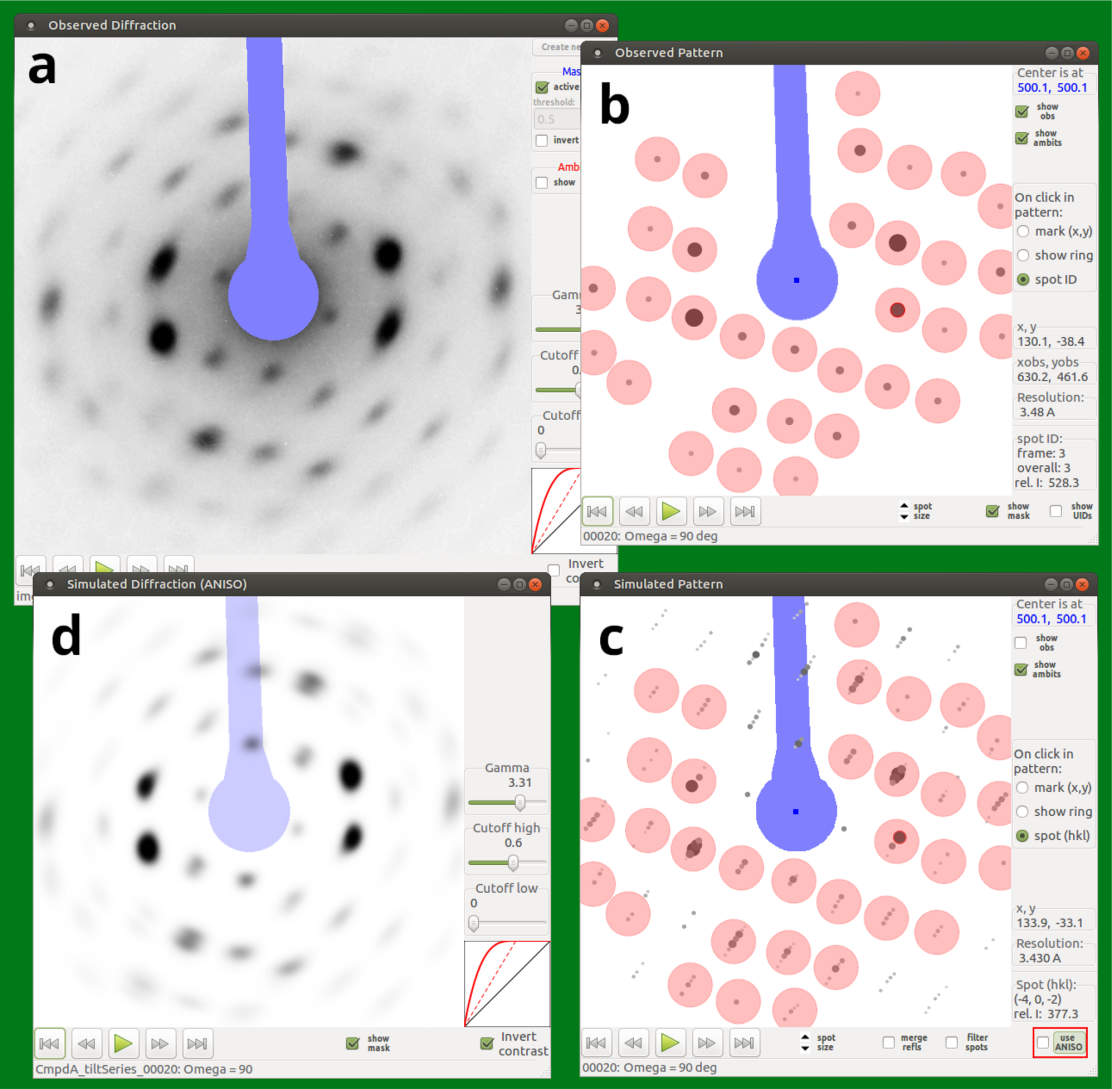
Types of diffraction images and patterns available from Garfield's Display menu. (a) Diffraction image with mask (blue, beamstop). (b) Diffraction pattern (schematic representation of the reduced data): Observed diffraction spots are symbolized by dark disks with sizes and gray levels decreasing with intensity; here, overlaid with red disks showing the spot ambits in use (see Sec. IV A for explanations). Weak diffuse reflections correspond to small disks of low density, which can be counter-intuitive. (c) Simulated pattern analogous to (b), with observed spots replaced by calculated Bragg reflections. Reflections within the same ambit can be merged to a single spot for easier comparison with (b). Simulation can be done with either standard or ANISO modeling (see Sec. IV D). As in the example case, there is often no significant difference between the results of the two methods. (d) Simulated diffraction image, calculated with the same parameters as used for (c). Simulation of diffraction images is always done with ANISO modeling (Figure prepared with data from Ishikawa *et al.*,^36^ HT phase of ${\text{Me}}_{4}P{\left[\text{Pt}{\left(\text{dmit}\right)}_{2}\right]}_{2}$).

The generation of temporally short and laterally confined high-fluence electron pulses inevitably leads to space charge effects that ultimately limit beam coherence.^35^ In order to mitigate these effects, the electron beam size at the sample in UED is usually quite large, typically on the order of 100 *μ*m in diameter. The combined requirement of a small sample thickness for high electron transmission and a large area to overlap with the beam dimensions makes UED samples susceptible to bending, cracking, corrugation, and other effects that degrade the sample quality and thus the quality of the diffraction data. Hence, most UED-ready crystalline samples are far from perfect single crystals and are better described as an ensemble of crystalline domains or crystallites with similar but not identical orientation. In addition, the preparation of UED samples, e.g., by thin-slicing crystals, often results in a high density of lattice defects, which reduces the average size of coherently diffracting domains and increases the effective size of reciprocal lattice points (RLPs) in all three spatial directions. For these technical and physical reasons, the Bragg reflections recorded in UED experiments are typically blurred and often overlap with other reflections, so that purely geometry-based indexing, which relies on precise peak positions, cannot be regularly applied. Typical UED data are of lower quality if judged from the viewpoint of well-established techniques of crystal structure analysis by x-ray or electron diffraction. An example from UED is shown in Fig. 1(a). For the determination of static crystal structures, such images are usually excluded from further consideration at an early stage of the standard workflows, e.g., in the “crystal screening” phase of single-crystal structure analysis or by fast and efficient filtering and restriction of indexing to “hits” in SFX and SSX. However, despite their shortcomings, time series of UED images can provide valuable information on structural dynamics, making the interpretation of such data highly desirable. Fortunately, the number of diffraction images to be indexed is usually small, namely, one per time series. Therefore, a different approach is required to overcome the challenges of indexing typical UED data.

The Garfield software package, which is the subject of this report, has been developed with the objective of facilitating the step of indexing single-crystal UED data under challenging conditions. In essence, the software simulates electron diffraction within a model that is computationally efficient and fast while retaining the relevant features found in typical UED diffraction patterns. The software provides tools for determining model parameters (including orientation of the crystal lattice) that, according to reasonable criteria, best describe the observed diffraction data. It differs from available indexing tools in several aspects. The most distinct differences are as follows: (1) the use of reflection intensities and peak positions in the analysis of diffraction data and (2) the use of a graphical user interface allowing direct and interactive control of the evaluation process by breaking out of the common data-in-result-out black-box approach. The Garfield approach to interpreting and indexing diffraction data is an interactive process. The objective is to identify the “best” solution, which is approached step by step. This involves excluding unlikely solutions and optimizing promising solutions through non-linear least squares (NLS) fitting of model parameters that describe diffraction geometry and sample properties. The two core tools in this process are GridScan, performing fast grid searches of possible orientations in three-dimensional rotation space, and GeoFit, a flexible tool for NLS fitting of individual parameters and whole parameter sets with presently up to 22 parameters for a single diffraction pattern. These include the (mean) orientation of the crystal lattice, the beam center, intensity and spatial scaling, sample thickness, and the orientation distribution of crystal domains.

The paper is structured as follows: Sec. II formulates the two guiding principles used to design the Garfield software package and describes how these principles are reflected in the chosen approach for indexing typical single-crystal UED data. Section III summarizes the technical requirements for installing Garfield (A), provides some general instructions and recommendations for working with the graphical user interface (B, C), and introduces the core tools, GridScan (D) and GeoFit (E). Section IV describes the underlying model used to predict the position and intensity of diffraction spots for a given set of model parameters. In Sec. V, the essential characteristics of Garfield are summarized, its benefits and limitations are discussed, and ways to improve its performance are outlined.

Although Garfield was developed with UED applications in mind and tested with data from low-symmetry molecular crystals, it can be useful in a broader range of applications where conventional methods for indexing fail. Additional examples of the application of Garfield can be found in the supplementary material.^73^

## II. DESIGN PRINCIPLES OF GARFIELD

Indexing single-crystal diffraction data is primarily a task of unraveling the geometric relationship between the probe beam direction, crystal lattice orientation, and detector geometry, with challenges posed by imperfect beam and crystal conditions coupled with detector insufficiencies. Conventional approaches rely on the precise determination of the positions of the recorded Bragg peaks, whereas reflection intensities are mostly disregarded, except for the precise localization of reflections and the discrimination of proper reflections from artifacts by considering peak profiles (post-refinement is outside of scope in the present context). If the unit cell parameters are known and accurate positions of the Bragg peaks have been extracted from a diffraction image, the orientation of the crystal lattice can in many cases be calculated within a fraction of a second by using one of the readily available indexing tools.

Since UED experiments typically lack precise information about the position of individual Bragg peaks, Garfield's first design principle (P1) is to use as much of the available information as possible. Hence, the discriminating information in reflection intensities should not be ignored. However, the application of this principle has consequences that go well beyond the inclusion of intensity data, as discussed below. The second principle (P2) requires that the fuzziness of recorded diffraction data (due to imperfections of the sample, for instance) should be taken into account in the process of finding the best interpretation of the data. First, it can provide additional discriminating information, and second, it allows the (mean) orientation of the crystal lattice to be fitted with standard least squares methods, which would not be possible for diffraction of a perfect crystal of infinite size assuming ideal scattering geometry.

Garfield's basic input must be provided as a list of detected diffraction coordinates $\left(x_{i_{o}}^{\text{obs}},y_{i_{o}}^{\text{obs}}\right)$ and partial intensities $I_{i_{o}}^{\text{obs}}$ (hereinafter called *reduced data*) as well as a crystal structure file in the CIF format.^37^ Hence, the program can only be used if the crystal structure (of the unperturbed crystal) is already known. Garfield calculates predictions $\left(x_{i_{o}}^{\text{clc}},y_{i_{o}}^{\text{clc}}\right)$ and $I_{i_{o}}^{\text{clc}}$ within a simple model in kinematical approximation ( $i_{o}=1,\ldots \;N_{\text{obs}}$, where $N_{\text{obs}}$ is the number of Bragg spots,^38^ or more precisely, the number of rows in the list of reduced data). In addition to physical and geometrical parameters (such as wavelength λ, detector distance, and rotation angles Θ,Φ,Ψ describing the orientation of the crystal lattice), the model includes parameters for quantifying several effects that affect the quality of recorded UED diffraction images: beam divergence, the finite size of the coherently diffracting domains, and—most important for UED—the domain orientation distribution. The latter will be referred to as *mosaicity*, although this may be an extension of the usual meaning. Using the GeoFit tool, almost all parameters, including the orientation of the crystal lattice, can be optimized by simultaneous NLS fitting. This is possible because the cost function *S*, the function to be minimized (the sum of squared residuals, SSR, essentially),

$$S=S^{\left(I\right)}+w_{P}\;S^{\left(P\right)},\;(w_{P}\geq 0),$$ (1)

which for a perfect crystal under ideal conditions is a discontinuous function of the orientation parameters, is smoothed and becomes a continuous function under imperfect conditions as a consequence of the fuzziness due to various blurring effects. In Eq. (1), $w_{P}$ is a control parameter that weights the discrepancy between predicted and observed peak positions, $S^{\left(P\right)}$, relative to the intensity-dependent part $S^{\left(I\right)}$ of the cost function. Using NLS fitting of positions *and* intensities in determining the orientation of the crystal lattice is one of the characteristic features that distinguish Garfield from other tools.

NLS fitting of 20 or more parameters, individually or simultaneously, is challenging. Convergence is not guaranteed, and starting values not too far from the solution are required in order to avoid convergence toward a wrong local minimum. Unsupervised fitting is therefore not practical under such conditions. Hence, Garfield's workflow is not designed to work like a black-box which automatically returns a result when a set of input data is entered, but rather provides a workbench that allows the user to work with multiple parameter sets and interactively explore the parameter space until a satisfactory result is found. This is the second fundamental difference between Garfield and other indexing tools. In essence, Garfield displays and manages a table of parameter sets that can be modified and extended by the user. In the following, this table will be referred to as the “main table of settings,” or the *main table* for short. Garfield provides various tools to create and modify settings, either by manual editing, by least squares optimization of existing parameter sets (GeoFit), or by systematic screening of crystal lattice orientations (GridScan). The goal is to arrive at a setting that optimally accounts for the totality of the recorded diffraction information.

The quality of different parameter sets can be evaluated by SSR values and crystallographic R-factors (after NLS optimization), and by visual comparison of Garfield's predicted diffraction with the observed diffraction. Although not strictly quantitative, the importance of visual comparison cannot be overstated, since it is often the prediction of fine details in the recorded diffraction, not captured by the input list of reduced data, that makes a particular parameter set most convincing. This can be viewed as a special kind of cross-validation and is consistent with the first design principle mentioned above (P1), which would be violated if only the reduced data were considered.

## III. WORKING WITH GARFIELD

### A. Installing Garfield

Garfield consists of a collection of scripts written in the R programming language for statistical computing.^39^ R is a free software available for common computer platforms. Garfield needs installation of R and additional packages available from the Comprehensive R Archive Network (CRAN). Although most of the code is platform-independent, Garfield currently runs only on a Linux system with the GTK2 and Cairo libraries installed. Detailed step-by-step installation instructions are included in the documentation/user manual provided in form of a TiddlyWiki,^40^ the file GarfieldWiki.html, which is part of the Garfield distribution.

### B. Setting up a new project

A Garfield *project* is defined by (1) a table of reflection positions and intensities extracted from either a single diffraction image to be indexed, or a tilt series of diffraction images; (2) the crystal structure (in form of a CIF file) which must be known *a priori*; and (3), optionally, a diffraction image (one or several in the case of a tilt series) in TIFF, JPEG, or PNG format for visual comparison and verification. Here and in the following, the simplest case of a project for indexing a single diffraction image will be assumed. The generalization to multi-image projects, e.g., for tilt-series obtained by rotating the sample about an axis perpendicular the incident beam, is self-explanatory.

Although all calculations are based exclusively on the reduced data (1) and the crystal structure (2), and thus, diffraction images (3) are not strictly necessary for Garfield to work, it is strongly recommended to attach representative diffraction image files to a project (see Sec. II). Full functionality is only attained if a diffraction image has been assigned. For instance, if a diffraction image is assigned, it is also possible to define a mask in order to exclude data from unreliable areas of the detector, such as the shadow of a beamstop. Furthermore, the quality of the results can be easily cross-checked within Garfield by comparing measured with simulated diffraction images. Otherwise, visual comparison is only possible by means of *diffraction patterns* (in Garfield's parlance), i.e., by two-dimensional scatter plots showing only the positions and integrated intensities of (observed or calculated) Bragg spots.

In Garfield's terminology, which is also adopted in the accompanying documentation and the remainder of this report, a diffraction *pattern* is a two-dimensional graphical representation of the reduced data (“obs”) or the predicted version of them (“clc”), obtained by plotting circular disks of different size and gray level (to indicate the intensities $I_{i}^{\text{foo}}$) centered at coordinates $\left(x_{i}^{\text{foo}},y_{i}^{\text{foo}}\right)$, “foo” = “obs” or “clc.” In contrast, a diffraction *image* corresponds to a matrix of gray values representing the pixel-by-pixel distribution of diffraction intensities, as if recorded with a two-dimensional array detector. Garfield provides options to view the observed diffraction images or patterns and to compare them with simulated counterparts.

The main window of Garfield displays a list of parameter sets (“settings”) for simulating the diffraction data of the current project (see Fig. S1 in the supplementary material).^73^ If the current project has just been created, the list consists of a single line, representing a dummy parameter set. In this case, in order to start working with the new project, the user must first create a new parameter set by editing the default parameters and assigning them realistic or at least meaningful values. Most of the parameters (including the orientation of the crystal lattice) should be reasonably well known from the experimental setup, but sometimes this is not the case. Other parameters, such as the mosaicity and the average size and shape of the crystallites, are usually not known in advance and must be fitted to the data by using the GeoFit tool.

### C. The main window

The main window is used to list and manage a growing collection of parameter sets used to explore the parameter space in order to find the optimal description of the diffraction experiment. New parameter sets can be added by manually editing existing sets or by saving the results of GridScan and GeoFit runs.

A complete set consists of model parameters (Table I) and control parameters (Table II). The former describe the diffraction geometry and sample properties. Many of them can be fitted with GeoFit. Control parameters include binary flags and numerical parameters that define details of how diffraction images are predicted and fitted, such as the type of modeling to be used (“standard” or “ANISO,” see Sec. IV D), the resolution range, or the *ambit radius* (radius of circular areas around observed diffraction spots used in the definition of the cost function, see Sec. IV A), and whether all predictions or only matching ones (“hits”) should be considered (for further explanations see Sec. IV).

**TABLE I.** Model parameters.

Parameter (s)	Edit^a^	Fit^b^	Comment
Electron energy	√		in keV
Wavelength	√		λ
Divergence	√		half-angle of circular cone
Bandwidth	√		Δλ/λ
Pixel size	√		equal in X and Y (LAB coord.s)^c^
Detector distance	√		*L*, real distance sample−detector
Camera length	√		$L_{\text{eff}}$
Magnification	√	√	$L_{\text{eff}}/L$
Beam center (X, Y)	√	√	on the detector (in pixels)
Image rotation^d^	√	( √)	due to magnetic lens
Image distortion	√	√	elliptical correction (2 param.s)
Orientation	√	√	of crystal lattice (3 param's)
Mosaicity	√	√	orientation spread of domains^e^
Shape transform	√	√	approximated by Gauss function^f^
Scale factor		√	overall intensity scaling
B factor		√	overall B factor correction
Omega axis^g^	(√)		direction of tilt axis^h^
Scale corrections^g^		(√)	n−1 correc. factors for *n* images
Omega corrections^g^		(√)	n−1 angles for *n* images

^a^ Parameters to be set in the Edit menu; redundant values are updated automatically.

^b^ Parameters that can be fitted with GeoFit.

^c^ X, Y, Z: Cartesian coordinate system, Z in the direction of the electron beam.

^d^ Cannot be fitted in a single-image project.

^e^ Standard model: normal distribution (one parameter); ANISO model: covariance matrix (six parameters).

^f^ Approximation of the squared magnitude of the effective shape transform. Standard model: width ( σ) and direction (tilt from Z) of reciprocal lattice rods (three parameters); ANISO model: covariance matrix of a 3D normal function (six parameters).

^g^ Only available in multi-image projects (tilt series).

^h^ The tilt axis must be perpendicular to Z (one parameter).

**TABLE II.** Control parameters.

Parameter	Description
dmin, dmax	resolution range (upper and lower d-spacing in Å)
ambit	radius (in pixels) of the spot ambits
mask	boolean: apply masking of bad detector regions
target	flag: “I” or “sqrt(I)”, see Eq. (8)
Pos.weight	weight of position errors, see Eq. (1)
fPred.weight	wt of non-matching predictions, $=w_{2}$ in Eq. (7)
ANISO	boolean: ANISO (true) or standard model (false)
Tilt.use ^a^	boolean: if true, take tilt of relrods into account
K0.corr ^b^	boolean: if true, apply per-tilt intensity corrections
Omg.corr ^b^	boolean: if true, apply per-tilt angle corrections

^a^ Only used in standard modeling.

^b^ Only available in multi-image projects (tilt series).

Any setting can be selected as the “active setting” by double-clicking on a row in the main table. Only one setting can be “active” at a time. Most of the options available via drop-down menus can only be used with the active setting defined. The parameters of the active setting are used when simulating diffraction patterns and diffraction images (menu “Display”), when searching for possible crystal orientations (menu “GridScan”), or as starting values for NLS parameter fitting (menu “GeoFit”).

### D. The GridScan tool

Once a new setting with more realistic parameter values has been created by manual editing, it is advisable to compare the diffraction pattern predicted by this setting with the observed pattern. If the predicted pattern shows resemblance to the observed pattern, it might be possible to proceed directly to the final step of optimizing the parameters by NLS fitting with GeoFit. Often this is not the case, and for the parameter values that are not known precisely enough, better start values for GeoFit must be found. The most critical parameters are clearly the crystal orientation parameters, because even a small misalignment can completely change the diffraction pattern. Other parameters that are typically affected by large uncertainties, such as mosaicity and effective domain size, are less critical, because the diffraction changes continuously with these parameters.^41^

**FIG. 2.**
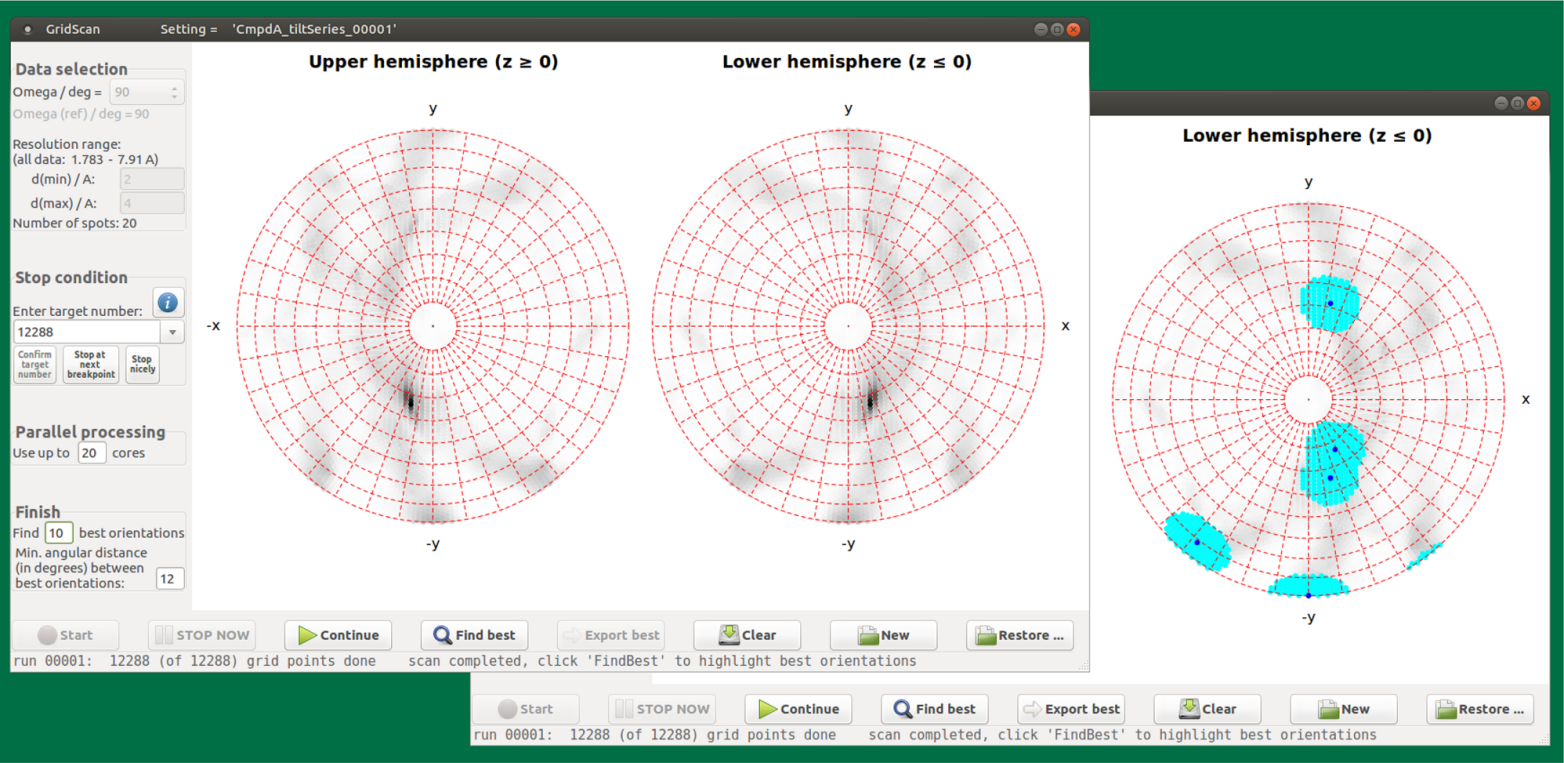
The GridScan window. All calculations in GridScan are performed with the parameters of the active setting, except for the resolution limits, d(min) and d(max), and the crystal orientation parameters, which are systematically varied so that the sampling of all possible orientations is as uniform as possible. The orientations are parameterized by the direction of the incident beam relative to the (fixed) crystal coordinate system, followed by a rotation of the crystal around the beam. A figure of merit (FOM) is calculated for each orientation, which measures the quality of the match between the predicted and observed diffraction pattern. The polar plots in the main part of the window show the maximum FOM found for each direction of the beam as density maps in equal-area Lambert projections of the upper and lower hemispheres. On the left margin, data used for GridScan can be filtered by restricting the resolution range (For a multi-image project, one image must be selected). The “resolution” (mesh size) of the grid to be searched is determined by the number of grid points tested (“target number”). Any number up to 49 152 (directions of the incident beam) can be entered. The computation can be stopped at any time (and continued later, if desired). Parallel processing is possible if multiple CPU cores are available. Foreground: FOM maps after testing 12 288 directions of the incident beam. Background: same as in foreground panel, with the 10 directions of highest FOM that are at least 12° apart from each other marked with a black dot in a cyan-colored surrounding.

The GridScan tool is an indispensable aid for finding better candidates for the orientation of the crystal lattice by performing a grid search over the entire SO(3) rotation space.^42^ When the tool is activated, a new window opens for setting up, starting, stopping, and monitoring the progress of grid searches (Fig. 2). In each run, the parameters of the active setting (the one that was active when GridScan was launched), except the orientation parameters and the resolution range, are used to compute a figure of merit (FOM)^43^ for up to 17 694 720 ( =49 152×360) orientations, by comparing the predicted patterns with the “observed” pattern.

In Garfield, the orientation of the crystal is defined by two polar angles, Θ and Φ, which define the direction of the incident beam relative to the crystal lattice, and a third angle, Ψ, for the rotation of the crystal around the incident beam.^44^ The grid is constructed from 49 152 predefined directions of the beam (Θ,Φ), each one combined with 360 rotations sampling Ψ in steps of 1°. The beam directions and the order in which they are visited are defined according to the principles of HEALPix (Hierarchical Equal Area isoLatitude Pixelation^45^). This sampling results in a most uniform and efficient^46^ coverage of the sphere with an angular resolution of 0.92°. The angular distance of any arbitrary orientation from the nearest grid point is less than 1°.

For each pair (Θ,Φ), represented by a point on the unit sphere, the maximum value of the FOM obtained by scanning over Ψ is plotted in two-dimensional gray-scale maps showing the projections of the upper and lower hemispheres onto the *x* and *y* plane.^47^ Good candidate orientations will show up as dark spots in these maps. On a modern multi-core workstation, a full grid scan takes only a few minutes to half an hour, depending on the number of diffraction spots used (typically 50–100). This number depends on the resolution range used for the grid scan, which can be set in the GridScan window before starting a scan. Also, the number *N* of beam directions to evaluate has to be set before starting a run ( N≤49152). A running grid scan can be aborted at any time (and resumed or extended at a later time, if this seems promising). Once a grid scan is finished (or has been terminated), the *n* best orientations on the FOM ranking can be exported to Garfield's main table, creating *n* new settings.

### E. The GeoFit tool

After creating a new setting (either by manual editing or using GridScan), it is usually necessary to optimize the current parameters by NLS fitting using the GeoFit tool (for more details, see the supplementary material, Fig. S2).^73^ The last step in a project should always be to run GeoFit to optimize the parameters and get a summary of the final results.

**FIG. 3.**
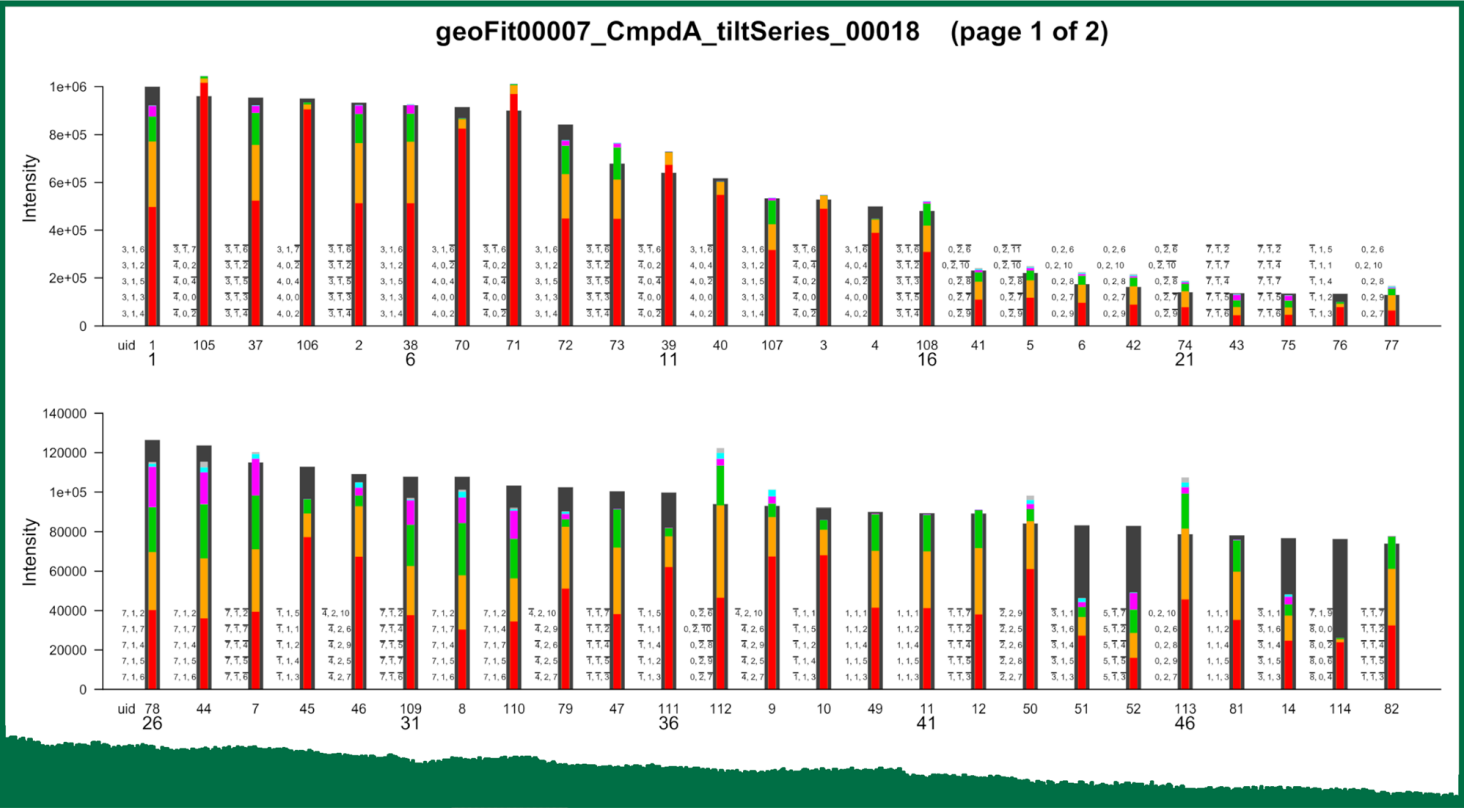
Observed spot intensities and the contributions of individual Bragg reflections according to GeoFit. Top part of the first page of the graphical output (pdf file) created with GeoFit run 00007 of project “CmpdA-tiltSeries,” starting from the parameters of setting 00018. The spots are sorted in descending order of observed intensities. The observed intensities are represented by black bars, and the calculated values by colored bars in front of the black bars (mostly obscuring the black bars). The length of the colored bars is divided in up to six segments proportional to the relative contributions of up to five individual Bragg reflections. The contributions are sorted from strongest (bottom) to weakest (top). If more than five reflections are contributing, a sixth segment (gray) representing the sum of the remaining contributions appears on top of the other segments. The Laue indices of the five strongest reflections are written next to the bars in the same order from bottom to top. The small numbers below the bars are generated unique identifiers of the observed Bragg spots.

The fitted values of all model parameters and the control parameters used for fitting can be saved for further use. Saving the results creates a new setting and adds a new entry to the Garfield's main table. In addition, several files are written to the file system for documentation, including (1) a listing of all parameters with their values before and after fitting, (2) a corresponding list of various kinds of SSR values and R factors, and (3) a comprehensive table of data related to Bragg reflections, combining the input coordinates and intensities of observed Bragg spots, the corresponding calculated intensities and centroid positions, and—for each spot—the proposed reflection indices *hkl*, expected intensities, and excitation errors (distances from the Ewald sphere) of up to five Bragg reflections contributing to the Bragg spot. A pdf file with a graphical representation of the most relevant information contained in the table (3) is also generated (see Fig. 3).

Note that in the example of Fig. 3, none of the observed spots is caused by a single Bragg reflection, but all spots are superpositions of multiple reflections. By considering spots with a dominant and one or several minor contributions, two extreme cases can be distinguished: (i) The structure factors are comparable in magnitude, but the excitation errors of the minor contributions are large compared to the dominant reflection. (ii) The excitation errors are comparable, but the structure factors are very different in magnitude. In case (i), but not in case (ii), it may be justified to neglect the contribution of spurious reflections to the temporal intensity variations measured in UED experiments.

## IV. PREDICTION OF DIFFRACTION PATTERNS

The purpose of the two core tools of Garfield, GridScan and GeoFit, is to find model parameters that reproduce the input data, the positions, and intensities of recorded Bragg spots (the reduced data). Bragg spots are either individual Bragg reflections or superpositions of them. In Garfield's terminology, the term diffraction pattern refers to the graphical representation of the reduced data. Thus, the two terms are essentially equivalent.

Predicting a diffraction pattern for comparison with the observed pattern requires (1) a crystallographic model describing the diffraction by the sample, and suitable algorithms to (2) estimate the expected diffraction and to (3) convert the result into a list of triplets $\left(x_{i_{o}}^{\text{clc}},y_{i_{o}}^{\text{clc}},I_{i_{o}}^{\text{clc}}\right)$ analogous to the input list of reduced data $\left(x_{i_{o}}^{\text{obs}},y_{i_{o}}^{\text{obs}},I_{i_{o}}^{\text{obs}}\right)$, where index $i_{o}=1,\ldots \;N_{\text{obs}}$ enumerates the observed Bragg spots. In order to be of practical value for NLS fitting of the model parameters, the model must be simple to allow fast computation. Also, the focus is on diffraction patterns (rather than diffraction images) because the algorithms computing the cost function in NLS fitting should be as fast as possible.

The potential loss of precision of the predicted spot positions $\left(x_{i_{o}}^{\text{clc}},y_{i_{o}}^{\text{clc}}\right)$ due to various simplifications and approximations in the model is compensated by additional restraints imposed on the model by considering the spot intensities as input data. The goal is to find the solution corresponding to the global minimum of the cost function. Taking intensities into account increases the contrast between different solutions, even if the calculated intensities $I_{i_{o}}^{\text{clc}}$ suffer from large uncertainties, but the correlation between estimated and observed intensities is positive. If a well-separated solution can be identified, the orientation of the crystal lattice is usually quite robust to errors in the calculated intensities, so that the assignment of Laue indices is not affected. Thus, the effect of considering intensities can be understood as the application of a filter that eliminates unlikely solutions. The effect of intensity uncertainties is to reduce the selectivity of the filter, rather than to bias the solution toward a completely different set of parameters, i.e., a solution that would produce a wrong indexing.

The following subsections describe how the above requirements (1–3) are implemented in Garfield. We start with (3), explaining how the predicted diffraction pattern is derived from the simulated diffraction. This provides an opportunity to introduce the concept of *spot ambits*, which is essential for understanding the whole approach and helps to put the limitations of the diffraction model into the right perspective. This first subsection (A) is followed by the definition of the cost function used in NLS fitting (B). The last two subsections describe the model itself (C) and how it is used to simulate the diffraction (D).

### A. From the diffraction to the diffraction pattern

In the kinematical theory of diffraction, a perfect crystal in a parallel, monochromatic beam will diffract only in well-defined directions and only if the crystal is oriented so that at least one point of the reciprocal lattice (in addition to the origin) fulfills the Bragg condition by intersecting with the Ewald sphere. If the crystal consists of small domains with slightly different orientations and the beam is not perfectly coherent, RLPs near the Ewald sphere can also give rise to diffraction. This can be viewed as an expansion of the RLPs from points of zero extent to distributions over a finite volume. When treated as distributions in reciprocal space, virtually all RLPs (up to a certain resolution) can contribute to diffraction, but most of them, however, with an intensity that is zero or near-zero.

For now, let us assume that the contributions of all RLPs, enumerated by their Laue indices *hkl*, have been calculated (as described in Secs. IV C and IV D) and are available as a table of centroid coordinates, integrated intensities, and corresponding Laue indices: $x_{j_{p}},\;y_{j_{p}},\;I_{j_{p}}$, and ${\left(\text{hkl}\right)}_{j_{p}}$ with $j_{p}=1,\ldots \;N_{p}$. The number $N_{p}$ of predicted reflections is usually much larger than the number of observed spots $N_{\text{obs}}$.

Since both the observed spot positions and the calculated predictions may be subject to ambiguity and uncertainty, a circular disk in the detector plane^48^ around $\left(x_{i_{o}}^{\text{obs}},y_{i_{o}}^{\text{obs}}\right)$ is defined as a neighborhood $A_{i_{o}}$ for each observation $i_{o}$,

$$A_{i_{o}}=\left\{(x,y)\;|\;{\left(x-x_{i_{o}}^{\text{obs}}\right)}^{2}+{\left(y-y_{i_{o}}^{\text{obs}}\right)}^{2}<r_{a}^{2}\right\},$$ (2)

and predictions $j_{p}$ are taken as contributions to spot $i_{o}$ if their centroids are within the spot's neighborhood, $(x_{j_{p}},\;y_{j_{p}})\in A_{i_{o}}$. The disks $A_{i_{o}}$ of radius $r_{a}$ will be referred to as the *ambits*^49^ of the observed spots. By default, $r_{a}$ is the maximum possible radius with no overlapping ambits, such that any point is either element of exactly one ambit or not element of any $A_{i_{o}}$.

Thus, each prediction $j_{p}$ is assigned to exactly one observed spot $i_{o}$ or none. For convenience, we define an index set $J=\left\{1,\ldots \;N_{p}\right\}$ and disjoint subsets of *J*,

$$J_{i_{o}}=\left\{j_{p}\in J\;|\;(x_{j_{p}},\;y_{j_{p}})\in A_{i_{o}}\right\}\;\forall i_{o}\in \left\{1,\ldots \;N_{\text{obs}}\right\},$$ (3)

as well as a set $J^{\text{nohits}}$ pointing to the predictions that do not fall within the ambit of any observed spot

$$J^{\text{nohits}}=J\setminus \underset{i_{o}}{\cup }J_{i_{o}}.$$ (4)

Combining all predictions contributing to a given observation means addition of the intensities and taking the weighted average of the centroid coordinates. Thus,

$$I_{i_{o}}^{\text{clc}}=\sum_{j_{p}\in J_{i_{o}}}I_{j_{p}},$$ (5)

$$x_{i_{o}}^{\text{clc}}={\left(I_{i_{o}}^{\text{clc}}\right)}^{-1}\sum_{j_{p}\in J_{i_{o}}}x_{j_{p}}\;I_{j_{p}},\;\text{if}\;J_{i_{o}}\neq Ø,\;(y_{i_{o}}^{\text{clc}}\;\text{alike}).$$ (6)

For quick comparison with the reduced data (the input diffraction pattern), Garfield provides the option to calculate the values in Eqs. (5) and (6) for any setting in the main table and to display the result as simulated diffraction pattern.

### B. The cost function

The parameters of a selected setting can be fitted with the GeoFit tool by NLS minimization of a cost function *S*, which is defined as the sum of squared differences between calculated and observed quantities that are related to the intensities and coordinates of diffraction spots [cf. Eq. (1)]. The exact form of the cost function can be adjusted by using a set of control parameters. One of the control parameters is $w_{P}$ already introduced in Eq. (1). Other control parameters are the minimum and maximum d-spacing of the resolution range to be considered.^50^

Two control parameters that should be mentioned are the flag target and another weighting factor, $w_{2}$. The first takes character string values “ I” or “ sqrt(I)” and determines whether the residuals based on intensities are calculated from the intensities directly or from the square roots of intensities. Essentially, this allows the user to change the weighting scheme used for the intensity dependent part $S^{\left(I\right)}$ of the cost function. The second, $w_{2}$, determines whether predicted reflections that are not within the ambit of any observation (most often “false predictions”) are simply ignored ( $w_{2}=0$, the default), or fitted toward zero intensity ( $w_{2}>0$). Positive values of $w_{2}$ should be used with caution, and only when GeoFit is trapped in a local minimum and tries to improve the fit of the observed intensities by generating many false reflections outside the ambits of all observed spots. Thus,

$$S=S^{\left(I,\text{hits}\right)}+w_{2}\;S^{\left(I,\text{nohits}\right)}+w_{P}\;S^{\left(P\right)},$$ (7)

where the intensity part $S^{\left(I\right)}$ has been split into two contributions.

$$S^{\left(I,\text{hits}\right)}=\{\begin{array}{cc} \sum_{i_{o}}w_{i_{o}}{\left(I_{i_{o}}^{\text{clc}}-I_{i_{o}}^{\text{obs}}\right)}^{2}, & \text{if}\;\mathrm{target}=“I”\\ \sum_{i_{o}}{\left(\sqrt{I_{i_{o}}^{\text{clc}}}-\sqrt{I_{i_{o}}^{\text{obs}}}\right)}^{2}, & \text{if}\;\mathrm{target}=“\text{sqrt}(I)”. \end{array}$$ (8)

If $w_{2}>0$, the contribution to $S^{\left(I\right)}$ by the predictions that cannot be assigned to any observation is calculated analogously, by setting the corresponding $I^{\text{obs}}$ values to zero^51^

$$S^{\left(I,\text{nohits}\right)}=\{\begin{array}{cc} \sum_{j_{p}\in J^{\text{nohits}}}{\left(I_{j_{p}}^{\text{clc}}\right)}^{2}, & \text{if}\;\mathrm{target}=“I”\\ \sum_{j_{p}\in J^{\text{nohits}}}I_{j_{p}}^{\text{clc}}, & \text{if}\;\mathrm{target}=“\text{sqrt}(I)”. \end{array}$$ (9)

The $w_{i_{o}}$ in Eq. (8) are individual weights, which in principle should be read from the input list of reduced data. However, weights (or sigma values) assigned to observed intensities are not taken into account in the present version of Garfield. Instead, the weights are determined as $w_{i_{o}}={\left(I_{i_{o}}^{\text{obs}}\right)}^{-\frac{1}{2}}$, which has the effect to weighing down high intensities relative to low intensities, similar to the effect of setting target=“sqrt(I)”.

The position part of the cost function is defined as the sum of squared distances between predicted and observed spot positions, $d_{i_{o}}^{2}={\left(x_{i_{o}}^{\text{clc}}-x_{i_{o}}^{\text{obs}}\right)}^{2}+{\left(y_{i_{o}}^{\text{clc}}-y_{i_{o}}^{\text{obs}}\right)}^{2}\;\text{if}\;J_{i_{o}}\neq Ø,\;r_{a}^{2}\;\text{otherwise}$. Thus,

$$S^{\left(P\right)}=\sum_{i_{o}}d_{i_{o}}^{2}.$$ (10)

It should be noted that position information present in the reduced data enters the cost function via the ambits, even if $w_{P}=0$ (the default) and, thus, $S^{\left(P\right)}=0$.

### C. Electron diffraction by typical “UED crystals”

All simulations in Garfield are calculated within the kinematical theory of diffraction. Multiple scattering, which can pose a serious problem for structure determination by electron diffraction, is ignored. For nearly perfect crystals, the dynamical theory of diffraction can in principle be used to cope with the effect of multiple scattering. This is a much more difficult (though not impossible^52^) task for typical samples used in transmission UED, where the sample quality is less perfect due to the reasons described in the Introduction. Thus, the application of dynamical theory in Garfield is not practical due to the enormous computational effort that would be required, and it is questionable whether the results would improve significantly.

Fortunately, the method used in Garfield for indexing UED patterns can tolerate quite large errors in the estimated diffraction intensities, as explained at the beginning of this section (Sec. IV). Furthermore, there are solid reasons to assume that dynamical effects are less pronounced in highly textured crystal, i.e., when the coherently diffracting domains are small and the mosaic spread is large^53,54^ as is often the case due to the typical limitations of UED experiments. This has recently been confirmed by simulations and comparison of electron diffraction calculated in kinematical and dynamical theory.^52^ According to the numerical results for single-crystal gold, application of the dynamical diffraction theory was indispensable for the accurate determination of the lattice temperature. However, even in this extreme case (closely packed heavy atoms) the errors of the kinematical intensities, measured by the R factor between kinematical and dynamical simulation, did not exceed much the 30% level, except for rather small tilt spreads ( $\sigma_{\theta }<20\;\text{mrad}$). An R value of 30% indicates that kinematical intensities are subject to large errors, but they nevertheless contain useful information that Garfield will exploit to determine the correct indexing.

Ignoring dynamical diffraction effects reduces the description of electron diffraction to a mere problem of considering the relative phases of waves scattered by the distribution of matter in the crystal, in strict analogy to x-ray diffraction. This leads to the Bragg or Laue conditions of diffraction, which can be visualized with the Ewald sphere construction. The transition from x-ray to electron diffraction is then essentially a matter of replacing x-ray atomic scattering factors with electron scattering factors and taking into account that for electrons, the radius of the Ewald sphere, 1/λ, is much larger than for X-rays and almost “plane-like.” Garfield uses the parameterizations of electron scattering factors of atoms and ions by Peng *et al.*^55,56^

The restriction to very thin samples relaxes the diffraction conditions to some extent, so that points of the reciprocal lattice can give rise to reflections even if the Laue conditions are not exactly fulfilled, provided that the *excitation error*, the distance of an RLP from the Ewald sphere, is not too large. Usually, this situation is interpreted in a different way: Instead of relaxing the Laue conditions, the RLPs are replaced by needle-shaped distributions parallel to the surface normal of the crystal sheet, and diffraction occurs, if a “reciprocal lattice rod” (*relrod*) intersects with the Ewald sphere. This is consistent with the idea, that the reciprocal lattice weighted with the structure factor represents the Fourier transform of a perfect crystal of infinite size, $F_{\infty }$, whereas the Fourier transform of a finite crystal is the convolution of $F_{\infty }$ with the Fourier transform of the crystal's shape function. For a plane-parallel plate of thickness *t* perpendicular to the incident beam, this leads to relrod profiles oscillating like a sinc function. The intensity measured for a particular RLP with indices *hkl* varies as ${\text{sinc}}^{2}(\pi \;t\;\Delta h)$ with the distance Δh of the RLP from the Ewald sphere (measured in units of $h=1/d_{\text{hkl}}$, the inverse of the d-spacing).

In UED experiments, the finite size of the crystal is not the only, and often not the most important reason why non-zero diffraction intensities occur for RLPs even if they are nominally not located on the Ewald sphere. Because of the large area of the diffracting volume (compared to its thickness) and the non-perfect quality of most samples, the orientation of the crystal lattice is not exactly the same in different parts of the sample. Thus, UED samples should rather be viewed as an ensemble of crystalline domains with slightly different orientations distributed around the average orientation of the crystal lattice. This is similar to the orientation distribution of mosaic blocks introduced by Darwin^57^ to quantitatively explain x-ray diffraction of single crystals. Although the orientation distribution in UED experiments can be much wider (typically several degrees) than the typical mosaic spread of good single crystals (usually ≪0.1°), the effect of orientation disorder will be referred to as *mosaicity* in the following. Other effects that contribute to the appearance of reflection spots in UED images are the divergence of the incident beam and, to a lesser extent, its finite energy bandwidth. In Garfield, all these effects are accounted for by following the example of the relrods introduced to explain the finite crystal thickness effect: RLPs (represented by Dirac delta functions) are replaced by smooth distributions, which should lead to correct results, at least approximately, if the Ewald sphere construction is used without modification.

To allow simple and fast computation of the combined effect, Garfield assumes normal distributions to describe the four effects. Gaussian approximations have been used in various formulations in serial snapshot crystallography to estimate reflection intensities and partialities (see Brehm *et al.*,^58^ and the references cited therein). In Garfield, the simplest case is assumed: each individual effect is described by a single Gaussian distribution, and the distributions are mutually independent. Then, the combination of any two effects corresponds to the convolution of two Gaussians, which in turn is a Gaussian distribution. Furthermore, the marginal and conditional distributions of multivariate Gaussians are also normal distributions. This allows a simple analytical formula to be used in many cases, while more realistic distributions would require time-consuming numerical integration. A few remarks are in order for all four effects.

#### 1. Finite size effect

**FIG. 4.**
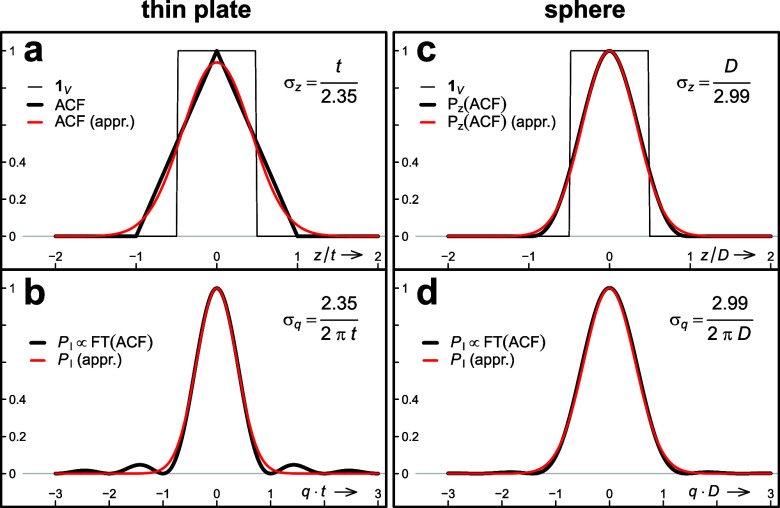
Autocorrelation function (ACF) and the intensity profile ( $P_{I}$) of the RLPs for a thin plate [(a) and (b)] and a solid sphere [(c) and (d)], and their approximation by normal distributions (red curves). (a) volume function ( $1_{V}$) and ACF along real space coordinate *z* perpendicular to a thin plate (thickness t≪ lateral extent); (b) $P_{I}$, the (normalized) Fourier transform of the ACF shown in (a), and the best approximation of $P_{I}$ by a normal distribution of the same height; (c) similar to (a) for a solid sphere of diameter *D*, except that the thick black line represents the (normalized) projection $P_{z}$ of the ACF to the *z* direction (arbitrary); (d) normalized Fourier transform of the ACF projection shown in (c) and best approximation by a normal function. $\sigma_{z}$ and $\sigma_{q}$ are the standard deviations of the normal distributions in real and reciprocal space, respectively. The numerical factors (2.35 for a thin plate, 2.99 for a sphere) were determined by minimizing the maximum of the absolute differences between $P_{I}$ and its approximation.

Given the fact that the intensity profile of the relrods for a thin plate [Fig. 4(b)] oscillates like the square of the sinc function, using normal distributions to describe the finite size effect may seem unphysical. However, the set of coherently diffracting domains (crystallites) of a typical UED sample varies not only in orientation but also in lateral extent and thickness. Averaging over crystallites of different thicknesses will fill in the minima of the intensity profile, $P_{I}(z)$, and results in smoothing, making the profile sufficiently normal. The smoothing will be even more pronounced if limitation of the domains in lateral directions (perpendicular to the beam) cannot be neglected. In this case, the relrods must be described by three-dimensional distributions of finite extension perpendicular to their main direction. An extreme case of diffraction by isometric particles is represented in Fig. 4(d) by the intensity profile for the solid sphere.

The intensity profile of the RLPs due to the finite size effect is described by a general three-dimensional normal distribution with six independent parameters: three standard deviations $\sigma_{i}^{\text{shp}}$ along the principal axes ( i=1,2,3) and three Euler angles. Degenerate distributions (one or two of the $\sigma_{i}^{\text{shp}}$ equal to zero) are allowed. Garfield is able to handle the case of a thin plate, all other broadening effects being negligible. However, due to the restriction to Gaussian profiles, the NLS fitting of thickness *t* is biased toward smaller values than the true average $\bar{t}$, since in this way the slow decay of the side maxima of the sinc function can be compensated by increasing the width of the Gaussian profile, $\sigma_{q}\propto t^{-1}$. In other words, Garfield cannot be used to fit the geometric thickness of the sample because the parameter t is correlated with, but not identical to, an effective thickness.

#### 2. Mosaicity

In contrast to the finite size effect, which is the same for all RLPs, the broadening of RLPs due to the orientation distribution of mosaic blocks^59^ increases linearly with the resolution, q=1/d. Here, *d* is the d-spacing, q=2 sinθ /λ, θ the Bragg angle, and λ the de Broglie wavelength of the electrons. Often the mosaicity can be quantified by a single angle, $\sigma_{\omega }$, the standard deviation of rotation angles ω between the mean orientation and the actual orientations of the mosaic blocks (or “domains”). This assumes that the mosaic spread is isotropic.

In typical UED experiments, a significant contribution to the mosaic spread is due to flatness imperfections resulting from the mechanical instability of thin plates and difficulties in sample preparation and mounting. Orientation distributions owing to such imperfections are not necessarily isotropic. For example, uniform bending of a flat plate would result in a systematic variation in local orientation (represented by the tangent planes), which is manifestly anisotropic.

Garfield accounts for the anisotropy of the mosaic spread by assuming that the rotation vectors $\vec{\omega }$, which represent the rotations of the domains from the mean orientation (averaged over the whole sample) to the actual orientation, are distributed according to a three-dimensional normal distribution.^60,61^ In general, this orientation distribution, $P_{\vec{\omega }}$, is described by six independent parameters, e.g., the standard deviations $\sigma_{i}^{\text{mos}}\;(i=1,2,3)$ along the principal axes and three Euler angles defining the orientation of the principal axes relative to the laboratory coordinate system (orthogonal axes *X*, *Y*, and *Z* with *Z* parallel to the incident beam).

The distribution $P_{\vec{h}}$ of the domains' reciprocal lattice vectors $\vec{h}$ induced by the rotations^62^ is determined by $P_{\vec{\omega }}$. Viewed as a three-dimensional distribution in reciprocal space, $P_{\vec{h}}$ is degenerate, as it occupies a two-dimensional surface given by a sphere of radius $h=|\vec{h}|$ centered at the origin. Other effects on the intensity profile set aside, diffraction conditions require to consider the intersection of this sphere with the Ewald sphere. Thus, diffraction intensity contributions due to mosaicity will appear as circular arcs. The intensity distribution along the arcs is defined by the conditional distribution obtained by restricting $P_{\vec{h}}$ to the (curved) line of intersection. From this, the expected intensity profiles on the detector can be derived, and their total intensities (integrated along the arcs) as well as the centroid positions can be calculated.

The exact calculation would be cumbersome and would require numerical integration of functions in three dimensions for all RLPs $\vec{h}=i\;{\vec{a}}^{⋆}+j\;{\vec{b}}^{⋆}+k\;{\vec{c}}^{⋆}$ of interest ( ${\vec{a}}^{⋆}$, ${\vec{b}}^{⋆}$, and ${\vec{c}}^{⋆}$ basis vectors of the reciprocal lattice). Garfield exploits the fact that $P_{\vec{\omega }}$ is a normal distribution (by assumption) and introduces an approximation which is only valid if all relevant rotation angles are sufficiently small. In the following, the approximation is first motivated by considering the case where all rotations are infinitesimally small; subsequently, the question is addressed as to how large the angles of rotation may be so that the approximation can still be used for practical purposes.

Assuming that the rotation vectors $\vec{\omega }$ (defining the orientation of the domains relative to the mean orientation) are normally distributed implies that $P_{\vec{\omega }}$ is a centered trivariate normal distribution of vector coordinates, for example, $\left(\omega_{x},\omega_{y},\omega_{z}\right)$, in the laboratory coordinate system. It is completely defined by its covariance matrix $\Sigma_{\vec{\omega }}$, which can be expressed in terms of the standard values and Euler angles mentioned above. In the non-degenerate case, its three-dimensional density function can be written as^63^

$$P_{\vec{\omega }}(\omega_{x},\omega_{y},\omega_{z})=\frac{\;\text{exp}\;\left\{-\frac{1}{2}(\omega_{x},\omega_{y},\omega_{z}){\left(\Sigma_{\vec{\omega }}^{\left(\text{xyz}\right)}\right)}^{-1}{\left(\omega_{x},\omega_{y},\omega_{z}\right)}^{⊤}\right\}}{\sqrt{{\left(2\pi \right)}^{3}\;|\Sigma_{\vec{\omega }}^{\left(\text{xyz}\right)}|}}.$$ (11)

**FIG. 5.**
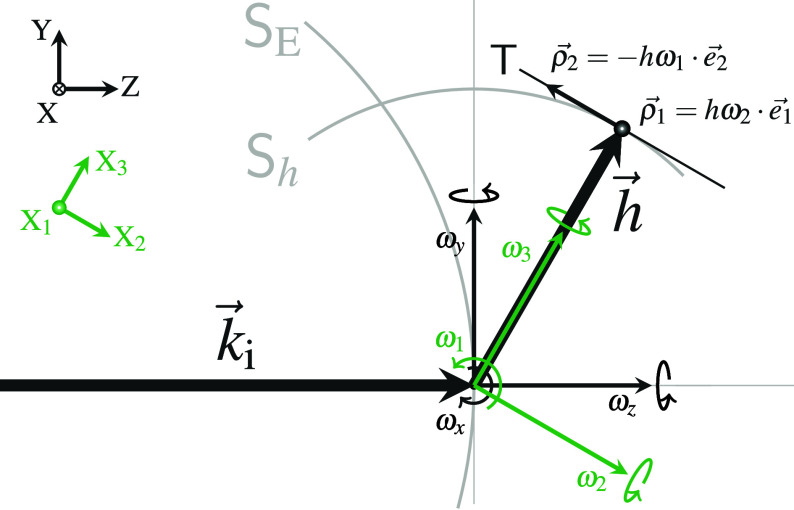
Effect of mosaic block rotation $\vec{\omega }$ on the position of an RLP and on the visibility of a corresponding Bragg reflection. $\vec{k_{i}}$ is the wavevector of the incident beam, $\vec{h}$ a vector pointing to an RLP in its unrotated (mean) position. $S_{E}$ is the trace of the Ewald sphere in the plane containing $\vec{k_{i}}$ and $\vec{h}$. The mosaic spread, described by a distribution of rotation vectors, leads to a distribution of RLPs over the sphere $S_{h}$ of radius $\left|\vec{h}\right|$ around the center. The diffracted intensity is proportional to the fraction of rotated vectors $\vec{h^{\prime }}$ that point to the intersection of $S_{h}$ and $S_{E}$. For each $\vec{h}$, a special Cartesian coordinate system $\left(X_{1}X_{2}X_{3}\right)$ is defined with unit vectors $\vec{e_{3}}\;||\;\vec{h}$, $\vec{e_{1}}\;||\;\vec{k_{i}}\times \vec{h}$, and $\vec{e_{2}}=\vec{e_{3}}\times \vec{e_{1}}$. Small (infinitesimal) rotations $\vec{\omega }$ with coefficients $\left(\omega_{x},\omega_{y},\omega_{z}\right)$ in the laboratory system (XYZ) and coefficients $\left(\omega_{1},\omega_{2},\omega_{3}\right)$ in $\left(X_{1}X_{2}X_{3}\right)$ are equivalent to three rotations around the axes of the $\vec{h}$-related coordinate system, $\vec{\omega_{i}}=\omega_{i}\cdot \vec{e_{i}}$ ( i=1,2,3). In tangent plane approximation, rotations $\vec{\omega_{1}}$ and $\vec{\omega_{2}}$ move the RLP by displacements $\vec{\rho_{1}}=\rho_{1}\cdot \vec{e_{1}}=h\omega_{2}\cdot \vec{e_{1}}$ and $\vec{\rho_{2}}=\rho_{2}\cdot \vec{e_{2}}=-h\omega_{1}\cdot \vec{e_{2}}$ within the tangent plane, T.

The first step in calculating the expected intensity profile of the reflection corresponding to a given RLP $\vec{h}$ is to transform the density function of $P_{\vec{\omega }}$ from the laboratory coordinate system to a local Cartesian coordinate system $\left(\omega_{1},\omega_{2},\omega_{3}\right)$ with the third axis parallel to $\vec{h}$. The other two axes are chosen to be perpendicular ( $\omega_{1}$) and parallel ( $\omega_{2}$) to the plane, which is spanned by $\vec{h}$ and the wavevector $\vec{k_{i}}∥Z$ of the incident beam ( $|\vec{k_{i}}|=1/\lambda$), see Fig. 5. The covariance matrix of the transformed density function will be denoted by $\Sigma_{\vec{\omega }}^{\left(123\right)}$.

If the rotation corresponding to $\vec{\omega }=(\omega_{1},\omega_{2},\omega_{3})$ is infinitesimal, it is equivalent to three consecutive rotations with angles $\omega_{1}$, $\omega_{2}$, and $\omega_{3}$ around the three axes of the coordinate system. Since rotation about the third axis does not move $\vec{h}$, the effect of $\vec{\omega }$ is the same as that of ${\vec{\omega }}_{⊥}=(\omega_{1},\omega_{2},0)$. Garfield assumes that the same holds, at least approximately, for all rotations of significant probability density. Then, $P_{\vec{\omega }}$ can be replaced by its marginal distribution, $P_{{\vec{\omega }}_{⊥}}^{⊥\vec{h}}$, obtained by integration over $\omega_{3}$. The result is a centered, bivariate normal distribution, the covariance matrix of which is obtained from $\Sigma_{\vec{\omega }}^{\left(123\right)}$ by dropping both the third row and third column

$$P_{{\vec{\omega }}_{⊥}}^{⊥\vec{h}}(\omega_{1},\omega_{2})=\frac{\;\text{exp}\;\left\{-\frac{1}{2}(\omega_{1},\omega_{2}){\left(\Sigma_{\vec{\omega }}^{\left(12\right)}\right)}^{-1}{\left(\omega_{1},\omega_{2}\right)}^{⊤}\right\}}{\sqrt{{\left(2\pi \right)}^{2}\;|\Sigma_{\vec{\omega }}^{\left(12\right)}|}}.$$ (12)

From the marginal distribution $P_{{\vec{\omega }}_{⊥}}^{⊥\vec{h}}$, a density function for the rotated $\vec{h}$ vectors can be derived straightforwardly. Within the infinitesimal rotations approximation, the distributions $P_{\vec{h}}$ and $P_{{\vec{\omega }}_{⊥}}^{⊥\vec{h}}$ are interchangeable, and the density function in Eq. (12) is also valid for $\vec{h}$ if variables $\left(\omega_{1},\omega_{2}\right)$ are interpreted as a special parameterization of $\vec{h}$.

An infinitesimal rotation $\vec{\omega }$ transforms an arbitrary vector $\vec{h}$ by adding an infinitesimal vector $\vec{\omega }\times \vec{h}$ to $\vec{h}$. Thus, in this approximation, the RLPs are distributed in a plane perpendicular to $\vec{h}$ (*tangent plane approximation*). The coordinates of the rotated vector corresponding to $\left(\omega_{1},\omega_{2}\right)$ are given by $\left(h_{1},h_{2},h_{3})=h(\omega_{2},-\omega_{1},1\right)$, where *h* is the length of the original $\vec{h}$ at mean orientation ( $\omega_{1}=\omega_{2}=0)$. Substitution of variables $\left(\omega_{1},\omega_{2}\right)$ by new variables $\left(\rho_{1}=h\;\omega_{2},\;\rho_{2}=-h\;\omega_{1}\right)$ in Eq. (12) yields

$$P_{\vec{h}}(\rho_{1},\rho_{2})=\frac{\;\text{exp}\;\left\{-\frac{1}{2}(\rho_{1},\rho_{2})\;\Sigma_{\vec{h}}^{-1}\;{\left(\rho_{1},\rho_{2}\right)}^{⊤}\right\}}{\sqrt{{\left(2\pi \right)}^{2}\;|\Sigma_{\vec{h}}|}},$$ (13)

with covariance matrix

$$\Sigma_{\vec{h}}=h^{2}\left[\begin{array}{cc} \Sigma_{22} & -\Sigma_{12}\\ -\Sigma_{12} & \Sigma_{11} \end{array}\right]$$ (14)

defined via the matrix elements of $\Sigma_{\vec{\omega }}^{\left(12\right)}=\left[\begin{array}{cc} \Sigma_{11} & \Sigma_{12}\\ \Sigma_{12} & \Sigma_{22} \end{array}\right]$.

In the tangent plane approximation, $\rho_{1}$ and $\rho_{2}$ can be identified with $h_{1}$ and $h_{2}$, the first two coordinates of $\vec{h}$ in the rotated coordinate system.

The expected intensity profile of the reflection corresponding to the RLP $\vec{h}$ can be obtained (up to a scaling factor containing the squared amplitude of the structure factor) by calculating $P_{\vec{h}}(h_{1},h_{2})$ along the trace of the Ewald sphere in the tangent plane. To a very good approximation, this trace is a straight line. The result is a normal distribution whose mean value (center position) and variance can be calculated from $\Sigma_{\vec{h}}$ by using another transformation of the coordinate system, such that the trace of the Ewald sphere is parallel to one of the new axes.

For non-infinitesimal rotations, the tangent plane approximation breaks down, and the curvature of the support of $P_{\vec{h}}$, i.e., the sphere of radius *h* around the origin, has to be taken into account. This is done in Garfield by applying geometric corrections that should perform well for all practical purpose.^64^ Hence, the most critical step in estimating diffraction intensities is the *infinitesimal rotations approximation* used for the transition from the original distribution $P_{\vec{\omega }}$ to its marginal distribution $P_{{\vec{\omega }}_{⊥}}^{⊥\vec{h}}$ and the identification of $P_{{\vec{\omega }}_{⊥}}^{⊥\vec{h}}$ with $P_{\vec{h}}$.

**FIG. 6.**
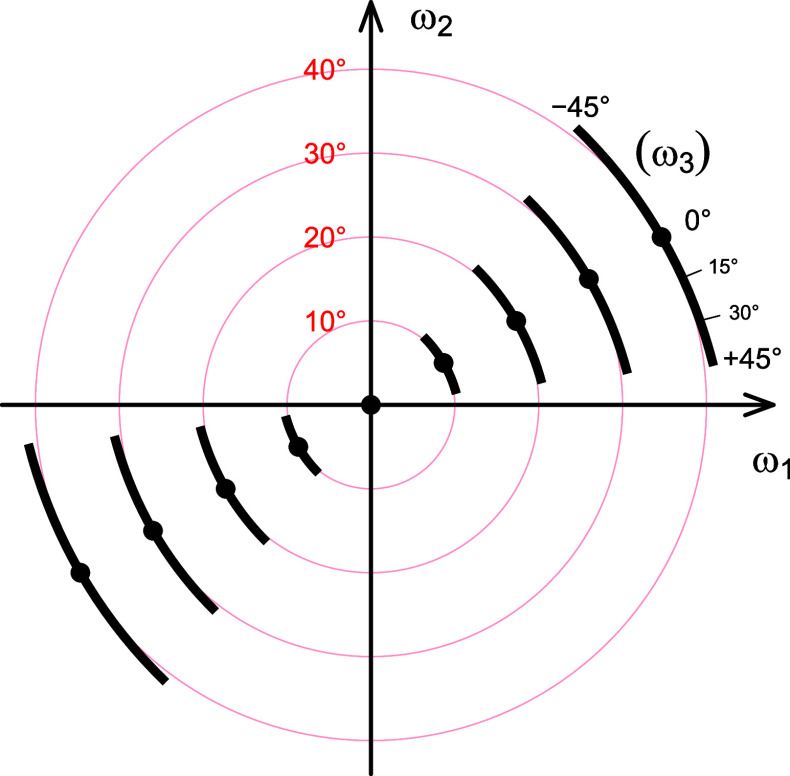
Error due to marginalization of $\omega_{3}$ at non-infinitesimal rotations. A couple of rotations with axes perpendicular to an RLP (with $\vec{h}∥\omega_{3}\;\text{axis}$) and rotation angles up to 40° are marked by a series of points in the $\left(\omega_{1},\omega_{2}\right)$ plane (black dots). The curved, nearly circular line segments centered at the dots are the projections of all rotation vectors with $\omega_{3}\in [-\pi /4,\pi /4]$ rotating $\vec{h}$ into the same direction (see the text for further explanations).

The limitations of this approximation are illustrated in Fig. 6. The density of the marginal distribution at any point of the $\left(\omega_{1},\omega_{2}\right)$ plane is obtained by integration of $P_{\vec{\omega }}$ along the $\omega_{3}$ axis, which is parallel to a certain RPL's $\vec{h}$ vector. However, this is not exactly what is required as rotation vectors $\left(\omega_{1},\omega_{2},0\right)$ and $\left(\omega_{1},\omega_{2},\omega_{3}\neq 0\right)$ do not move the RLP exactly to the same position, unless $\omega_{3}$ is infinitesimal. In order to integrate densities of rotations that move the RLP to identical positions, $P_{\vec{\omega }}$ should be integrated along curves such that all points of these curves correspond to rotations that result in the same position of the RLP. Projections of such curves to the $\left(\omega_{1},\omega_{2}\right)$ plane are shown in Fig. 6.

One observation that follows directly from looking at Fig. 6 is that the marginal distribution intermixes orientations ( $\vec{\omega }$ vectors) within angular ranges that increase almost linearly with $\omega_{3}$, leading to a certain alteration of the correct distribution. The figure also shows that intermixing of nonequivalent orientations has virtually no effect if the original distribution is isotropic, and that errors due to intermixing increase with the degree of anisotropy. Except in extreme cases of anisotropy, reducing the marginal distribution to a line in the $\left(\omega_{1},\omega_{2}\right)$ plane (for example the line through the black dots in Fig. 6), standard deviations $\sigma_{i}^{\text{mos}}$ up to 5° (i.e., 3 sigma < 15°) should be acceptable, considering that Garfield's requirements for the precision of position and intensity estimates are not very high. Even sigma values in excess of 10° may be tolerable if the anisotropy is moderate.

#### 3. Beam divergence

In Garfield, the angular spread of the incident wavevectors, ${\vec{k}}_{i}$, is modeled as a rotationally symmetric normal distribution with sigma values $\sigma_{x}=\sigma_{y}=\sigma^{\text{divg}}$. The effect of a finite beam divergence on the diffraction image is treated approximatively by splitting the effect in two parts and treating them separately. (i) The first effect is that more reflections can appear because more RLPs may “touch” the bundle of Ewald spheres corresponding to the distribution of ${\vec{k}}_{i}$ vectors, and the intensities of all reflections change according to the change of “mean” or “effective” excitation errors. (ii) The second part concerns the reflections' positions and profiles in the detector plane, which are affected by beam divergence due to the range of projection directions associated with the angular spread of the ${\vec{k}}_{i}$ vectors.

The first effect is taken into account by replacing the distribution $P_{\vec{\omega }}$ of orientation vectors (the “mosaicity”) by the convolution of $P_{\vec{\omega }}$ with the distribution describing the divergence of the ${\vec{k}}_{i}$ vectors.^65^ Thus, this part is treated by replacing the mosaicity with an “effective mosaicity,” while the Ewald sphere construction remains unchanged.

In the calculation of the cost function (for parameter fitting), the second effect is ignored. The cost function only depends on the center positions and integrated intensities of predicted reflections. The profiles are irrelevant, and the shift in center positions is negligible if the beam divergence is significantly smaller than the mosaicity, which is the case in typical UED experiments. Nevertheless, for simulating the diffraction *image*, the broadening of reflection profiles by $\sigma^{\text{divg}}$ is approximately taken into account.

#### 4. Energy bandwidth

An energy distribution around the mean electron energy leads to a distribution of $k_{i}=1/\lambda$ values and thus to a distribution of Ewald spheres with radii $k_{i}$. Again, Garfield uses a normal distribution (sigma value $\sigma^{\text{bwdth}}$) to describe the effect of a finite bandwidth. Similar to beam divergence, the energy bandwidth affects (i) which RLPs give rise to a reflection and with what intensity, and (ii) where in the diffraction image the reflections appear and what their intensity profile is. Garfield treats the effect of energy distribution in an approximative manner, by separating the two aspects. This leads to another increase in the effective mosaicity by convolution with the distribution describing the energy spread. In this case, however, the modification of $P_{\vec{\omega }}$ depends on the resolution, i.e., the distance of the RLP from the origin, $\left|\vec{h}\right|$. For the same reason as in the treatment of beam divergence, the second effect (ii) is considered only when simulating diffraction *images* (where reflection profiles should be as realistic as possible). For parameter fitting and predicting diffraction *patterns*, the second effect is ignored.^66^

### D. Further approximations and computational shortcuts

Using normal distributions to describe the four effects discussed in Sec. IV C has the advantage that predicting reflection intensities and positions is largely reduced to algebraic manipulations of covariance matrices. The combination of beam divergence and energy spread with the orientation distribution of coherently diffracting domains is not a major complication and can be achieved by convolution, resulting in an effective mosaicity. This is unproblematic because it describes the broadening of the RLPs' diffracting profiles due to three effects that are all of the same kind: All three are related to relaxing one of the conditions of an ideal diffraction experiment with scattering geometry defined by a unique direction of the incident beam, a unique energy of the electrons, and a unique orientation of the crystal lattice.

The finite size effect is of a different kind. Unless the (average or effective) shape function is spherically symmetric, it is not sufficient—in principle—to know $P_{\vec{h}}(\vec{h})$, the density of RLPs at any point $\vec{h}$, without also considering the orientation of the corresponding subset of crystallites, which deviates from the average orientation of the crystal lattice by virtue of $\omega_{1}=-\rho_{2}/h$, $\omega_{2}=\rho_{1}/h$, and $\omega_{3}$. Within the *tangent plane approximation*, rotation of the shape function (represented by normal distribution $P_{\text{shp}}$) due to $\omega_{1}$ and $\omega_{2}$ can be neglected. Thus, for this part, a simple convolution of $P_{\vec{h}}$ with $P_{\text{shp}}$ would suffice. Extending this approach to larger values of $\omega_{1}$ and $\omega_{2}$ should be possible by means of the geometric corrections mentioned in Sec. IV C 2.^67^

With regard to $\omega_{3}$, an exact treatment is computationally more expensive, since all information about $\omega_{3}$ rotations is lost when calculating $P_{\vec{h}}(\omega_{1},\omega_{2})$, the marginal distribution of $P_{\vec{\omega }}(\omega_{1},\omega_{2},\omega_{3})$. The exact treatment would involve three steps: instead of using $P_{\vec{h}}(\omega_{1},\omega_{2})$, consider the conditional distributions $P_{\vec{\omega }}(\omega_{1},\omega_{2}\;|\;\omega_{3})\;d\omega_{3}$, calculate their convolution with $P_{\text{shp}}$ rotated by $\omega_{3}$, and numerically integrate the results over $\omega_{3}$. We considered such a protocol, but the results did not justify the extra computational costs associated with it, and in the current version of Garfield, the rotation of the shape function due to $\omega_{3}$ is ignored.^68^

Convolution of the bivariate (degenerate trivariate) distribution $P_{\vec{h}}$ with the shape function results in a non-degenerate trivariate distribution. This poses no further problems. The result is a two-dimensional intensity profile with non-zero width in radial direction. The radial width is only determined by the shape function.

Anisotropy of the orientation distribution appears to be a common phenomenon in UED, but it is not always a dominant feature. Whenever possible, the general treatment described above should be avoided by using a simpler approach that permits much faster calculations by ignoring mosaic anisotropy. In the GeoFit tool and for simulating and displaying diffraction *patterns*, Garfield offers the option to choose between two methods for predicting position and intensity of reflections: the method presented in Sec. IV C and the previous paragraphs (“ANISO modeling”), and a simplified version called “standard modeling.”^69^

In standard modeling, the four factors that determine the RLPs' diffracting power profiles are all described by Gaussian functions with standard deviations $\sigma^{\text{shp}}$, $\sigma^{\text{mos}}$, $\sigma^{\text{divg}}$, and $\sigma^{\text{bwdth}}$. Here, the orientation spread measured by $\sigma^{\text{mos}}$ is assumed to be the same in all directions (i.e., isotropic), while the shape profile measured by $\sigma^{\text{shp}}$ is restricted to a certain direction (“relrods”). Standard modeling assumes that the coherently diffracting domains of the sample can be viewed as thin plates. Their finite extent in lateral directions is ignored.^70^

In order to estimate reflection intensities and positions in standard modeling, first, the effects associated with uncertainties in scattering geometry are combined, resulting in an effective mosaicity with variance ${\left(\sigma^{\text{MOS}}\right)}^{2}={\left(\sigma^{\text{mos}}\right)}^{2}+{\left(\sigma^{\text{divg}}\right)}^{2}+{\left(a\;h\;\sigma^{\text{bwdth}}\right)}^{2}$, where *a* is a constant and $h=|\vec{h}|$. Then, the effective mosaicity is combined with the shape profile to obtain the width (sigma value, $\sigma^{*}$) of the effective Gaussian profile of an RLP at $\vec{h}$,

$${\left(\sigma^{*}\right)}^{2}={\left(\sigma^{\text{shp}}\right)}^{2}+{\left(h\;\sigma^{\text{MOS}}\right)}^{2}.$$ (15)

**FIG. 7.**
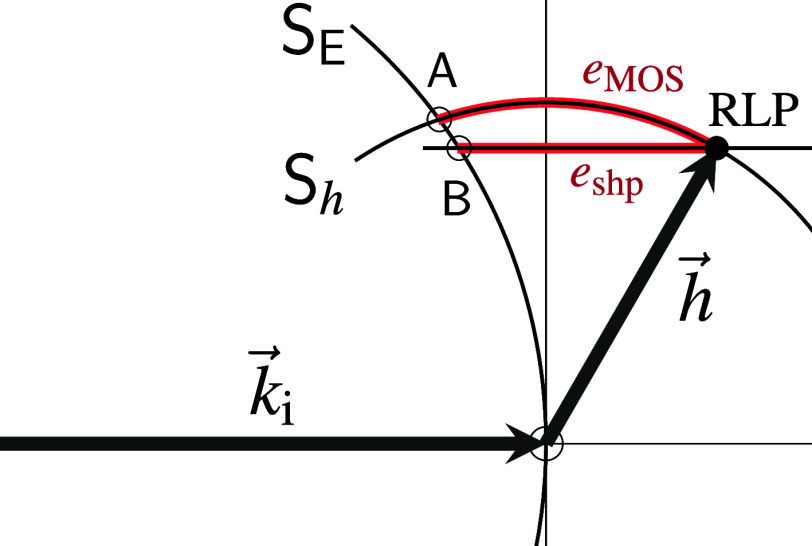
Ewald sphere construction to predict the position and intensity of a reflection belonging to an RLP at reciprocal lattice vector $\vec{h}$. $S_{E}$ is the trace of the Ewald sphere in the plane containing the RLP and the incident wave vector ${\vec{k}}_{i}$. Two extreme cases with intersection points A and B are shown, as discussed in the main text. The “excitation errors” $e_{\text{MOS}}$ and $e_{\text{shp}}$ are the distances of the RLP from $S_{E}$ measured along the circle around the origin with radius *h* (i.e., the trace of sphere $S_{h}$) and along the direction of the relrods, respectively. Note that the relrod in the figure is parallel to ${\vec{k}}_{i}$. This is a special case. In general, relrods can be tilted in any direction. Note also that in electron diffraction, the radius of $S_{E}$ is much larger than that of $S_{h}$.

Finally, $\sigma^{*}$ is used to estimate the integrated intensity and centroid position of the reflection associated with the RLP. Since $\sigma^{*}$ involves two fundamentally different effects, this cannot be done in a straightforward manner. Consider the two extreme cases: (1) $\sigma^{\text{shp}}\ll h\;\sigma^{\text{MOS}}$ and (2) $\sigma^{\text{shp}}\gg h\;\sigma^{\text{MOS}}$, as illustrated in Fig. 7.

(1) If broadening of the RLP profile due to the shape function can be neglected, the profile is determined by the (effective) orientation distribution, so $\vec{h}$ is spread over the surface of the sphere $S_{h}$ with radius *h* around the origin. Without introducing further assumptions, the best guess for the centroid position of the expected reflection corresponds to the point A on the intersection of the Ewald sphere with $S_{h}$ and the plane that is spanned by ${\vec{k}}_{i}$ and $\vec{h}$. Let $e_{\text{MOS}}$ be the distance from the RLP to A, measured along $S_{h}$ in this plane. Then the intensity can be estimated by the value of the profile at $e_{\text{MOS}}$ (up to a factor containing the structure factor amplitude squared).
(2) If the effective mosaicity is negligible compared to the finite size effect and the shape function is that of a thin plate, $\sigma^{*}$ represents the diffracting power profile of relrods, which are usually more or less parallel to the (mean) surface normal of the crystal foil. The expected centroid position of the reflection is then determined by the point B where the straight line through the tip of $\vec{h}$ parallel to the direction of the relrods hits the Ewald sphere. The estimated intensity is proportional to the value of the Gaussian density profile at $e_{\text{shp}}$, the distance between B and the tip of $\vec{h}$.

In standard modeling, Garfield calculates intensities and centroid positions for both extremes and takes a weighted average (with weights proportional to $\sigma^{\text{shp}}/e_{\text{shp}}$ and $h\;\sigma^{\text{MOS}}/e_{\text{MOS}}$) as estimate for the general case.

## V. CONCLUSION

Garfield is an interactive software package that helps to (A) find the *hkl* indices of Bragg reflections recorded in UED experiments and (B) to quantify the intensity contributions of individual reflections when several of them overlap to form a merged “Bragg spot.” The latter is a common challenge due to the high mosaicity of typical samples in UED. Often the orientation distribution dominates the effect of limited thickness, as is the case in the example of Fig. 1.

**FIG. 8.**
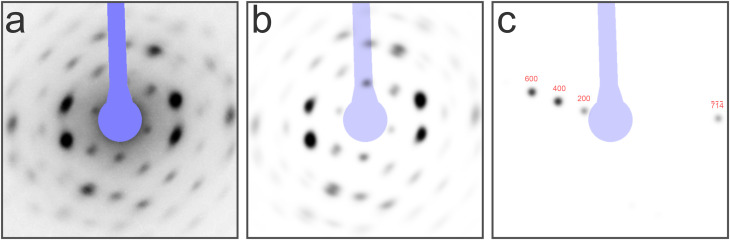
Importance of the orientation spread (mosaicity). (a) Recorded and (b) simulated diffraction images as in Fig. 1 [(a) and (d)]. The mosaicity was fitted with GeoFit to $\sigma_{1}=\sigma_{2}=4.7{}^{\circ}$ ( $\sigma_{3}=3{}^{\circ}$ was fixed, see main text). (c) Simulated image computed with the same parameters as in (b), except that the mosaicity was set to zero ( $\sigma_{1}=\sigma_{2}=\sigma_{3}=0$). Note that intensities are always scaled relative to the highest value. The reflections in (b) are also present in (c), but their contribution to the observed diffraction is very weak. For example, the spot near the reflection (400) is actually dominated by the (402) reflection (Figure prepared with data from Ishikawa *et al.*,^36^ HT phase of ${\text{Me}}_{4}P{\left[\text{Pt}{\left(\text{dmit}\right)}_{2}\right]}_{2}$; reflection (402) of the HT phase corresponds to ( $\bar{8}$06) of the low-temperature CS phase).

Neglecting the orientation spread would completely change the character of the expected diffraction, as demonstrated in Fig. 8. Of the large number of recorded reflections, only a few would be expected, and—what is even worse—the expected reflections are not the intense ones that really dominate the diffraction and largely determine the center position of the observed spots.

Garfield relies on approximations of the scattering process such as kinematic diffraction theory and the assumption of normally distributed quantities to mitigate computational costs when predicting reflection intensities from the values of a certain set of model parameters, as described in Sec. IV. These approximations are supported by physical arguments and have shown resilience in practical use cases.

To A: Like other indexing tools, Garfield uses the position of observed reflections to determine the orientation of the crystal lattice. The differences are due to the fact that in the cases envisaged for the application of Garfield, the accuracy of the position data cannot be trusted as much as in x-ray diffraction off high-quality single crystals. The uncertainty in reflection positions is taken into account by defining so-called “ambits” around the Bragg spots. The relaxation of positional constraints through the introduction of ambits can lead to difficulties in discriminating between two or more possible solutions. Fortunately, such ambiguities can often be resolved by also considering the predicted intensities, even if these intensities have large margins of error. As an additional quality check of the predictions, visual comparison of simulated and recorded diffraction patterns introduces an element of “cross-validation” that turns out to be crucial. If by all criteria, such as SSR values and R factors, plausibility check of the model parameters, and visual comparison of diffraction images, it is not possible to discriminate between nonequivalent solutions, the task must be considered unsolved.^71^

Even if in a particular physical situation the Bragg reflections do not overlap—such that single triplets of Laue indices *hkl* can be assigned to each Bragg spot—finding the correct indexing for a single diffraction image can still be a challenge (for any tool) if nonequivalent lattice planes have identical or similar metrics by pseudo-symmetry or by accident. Working interactively with Garfield, especially when using GridScan, increases the chance of becoming aware of potential pitfalls. Using reflection intensities as additional discriminating information will in most cases favor the correct solution even if the intensity estimates are not very accurate.

To B: Superposition of two or more Bragg reflections in a single diffraction peak is not an uncommon situation in UED experiments. If such maxima are to be used to study ultrafast structural changes by pump-probe experiments, knowledge of the fractional contributions of individual reflections is crucial for the interpretation of measured intensity variations. In the case of overlapping reflections, Garfield provides not only reflection indices for all reflections involved, but also returns estimates of the individual intensity contributions to enable further analysis.

The validity of the approximations used to estimate individual reflection intensities is crucial if the relative contributions are to be used in the structural dynamics analysis. The same standards should be applied here as for structure analysis with electron crystallography in general. In particular, the sample must be thin enough so that effects of dynamical and multiple scattering are sufficiently suppressed. If this is not the case, Garfield's estimates of diffraction intensities are compromised. If dynamical scattering can be ignored, but the errors due to other approximations (e.g., the assumption of normal distributions to describe the orientation spread and diffracting power of RLPs) are inadequate, one option might be to find better estimates, for example by using a better model or by applying more precise calculations. Another possibility could be to separate the contributions of individual Bragg reflections through a detailed analysis of the spot profiles. Garfield's intensity estimates might be useful as start values for profile fitting.

As input, Garfield needs a list of measured diffraction spot intensities and corresponding coordinates. Information contained in spot profiles is not taken into account. The first step in the analysis of UED images, the extraction of intensities and positions from recorded images, must be done outside of Garfield and prior to using the program. So far, Garfield has little flexibility to ensure an optimal match between the way data were extracted from the recorded diffraction images and Garfield's particular way of using those data in parameter fitting and indexing: A subset of the input data can be selected by enabling or disabling a mask or by restricting the resolution range to use, and the default ambit radius can be changed if deemed advisable. For future releases of Garfield, data reduction tools (including background subtraction, masking of regions, definition and localization of Bragg spots, integration of spot intensities) and the tools for model fitting and indexing provided by the present version should both be made accessible via the same interface for enhanced user experience.

## Data Availability

Garfield is openly available as a collection of R scripts and related files from Edmond at https://doi.org/10.17617/3.CXELBR, Ref. 72.
